# A Systematic Review on the Impact of Hypofractionated and Stereotactic Radiotherapy on Immune Cell Subpopulations in Cancer Patients

**DOI:** 10.3390/cancers14215190

**Published:** 2022-10-22

**Authors:** Silvia Takanen, Marta Bottero, Paola Nisticò, Giuseppe Sanguineti

**Affiliations:** 1 Radiation Oncology, IRCCS Regina Elena National Cancer Institute, 00144 Rome, Italy; 2 Unit Tumor Immunology and Immunotherapy, IRCCS Regina Elena National Cancer Institute, 00144 Rome, Italy

**Keywords:** hypofractionated radiotherapy, stereotactic body radiation therapy, peripheral immune cells, lymphopenia, lymphocytes, neoplasms

## Abstract

**Simple Summary:**

The role of different radiotherapy fractionation on immune cells is yet to be determined. Monitoring immune cells and understanding their quantity and quality changes during and after radiotherapy could have relevant implications on the development of novel therapeutic strategies. The aim of our review was to analyze the evidence of the literature regarding radiation-induced changes in immune cells in patients undergoing stereotactic body radiation therapy or hypofractionated radiation therapy and assess their potential impact on future combined systemic therapies. Preliminary studies seem to confirm a strong modification of the tumor immune environment and peripheral immune cell landscape after hypofractionated and stereotactic regimens.

**Abstract:**

We investigated how hypofractionated radiotherapy (HFRT) and stereotactic body radiotherapy (SBRT) may impact immune cells in different type of tumors. A systematic review was performed according to the Preferred Reporting Items for Systematic Reviews and Meta-Analyses (PRISMA) guidelines. The PubMed, Embase and Cochrane databases were searched. Overall, 11 studies met the inclusion criteria and were eligible for the present analysis. Both HFRT and SBRT have different impact on lymphocyte subpopulations, confirming their immunomodulatory effect which may have a crucial role in future combined treatment with new emergent therapies such as immunotherapy. Further studies are needed to shed more light on this emerging topic to ultimately improve patient care, treatment and clinical benefits for cancer patients.

## 1. Introduction

Radiotherapy (RT) is part of the multidisciplinary management of cancer patients and along with surgery and systemic medical therapies, including immunotherapy, plays a key role in local tumor control.

Due to technical improvements and a better understanding of tumor radiobiology, hypofractionated RT (HFRT) (≥2 Gy/fraction) has been introduced in recent decades for different types of tumors to exploit the administration of higher doses per fraction in a shorter period of time. Stereotactic body radiation therapy (SBRT) is a further evolution of HFRT in which the overall treatment is condensed in few fractions of even higher dose per fraction (≥5 Gy/fraction). In order to be safe, the dose has to be tightly conformed to the target, implying a drastic reduction of the dose to the surrounding organs at risk [1].

RT modulates the host immune response, but the direction of the effect varies greatly among different tumor types. Indeed, RT can determine either an immune-stimulatory effect, inducing a tumor-specific immune response, or an immune-suppressive effect by increasing the expression of immunosuppressive molecules, such as the upregulation of the expression of programmed death domain ligand-1 (PDL-1) and cytotoxic T lymphocyte antigen-4 (CTLA-4) [2].

Lymphocytes are key effector cells of the immune system and their absolute count (ALC) is often reduced after RT, resulting in a phenomenon known as radiation-induced lymphopenia (LP). Pre- and post-treatment LP is also associated with chemotherapy or other cancer related therapies [3], representing an adverse prognostic factor for progression-free survival (PFS) or overall survival (OS) in a wide variety of tumors [4]. Radiation treatment modality has been shown to play an important role in RT-induced LP [5]. The reduction of both the irradiated volume and the number of treatment sessions through both SBRT and HFRT schedules could limit normal tissue exposure and, consequently, the risk of severe LP [6].

Therefore, it is not surprising that, compared to conventionally fractionated RT (CFRT), both HFRT and SBRT have been associated with a reduced absolute lymphocytes count (ALC), possibly leading to improved response and PFS rates [7].

While preserving patient immune status during RT is crucial to achieve a host response against the tumor, it remains unclear whether HFRT or SBRT may be associated with improved response rates and outcomes by eliciting different immune-related effects such as an enhanced lymphocyte preservation or increased antigen presentation, or both.

There are data in the literature pertaining to radiation-induced leucotoxicity and LP, although those covering more detailed changes in lymphocyte subtypes, especially after HFRT or SBRT, are scarce. Understanding and monitoring these changes during and after RT could have implications on the development of novel therapeutic strategies (e.g., immunotherapy + RT) [8].

The aim of this systematic review is to analyze the current evidence of the literature regarding radiation-induced changes in immune cells in cancer patients undergoing SBRT or HFRT and to assess their potential impact on future combined systemic therapies.

## 2. Materials and Methods

### 2.1. Search Strategy

**Figure 1:**
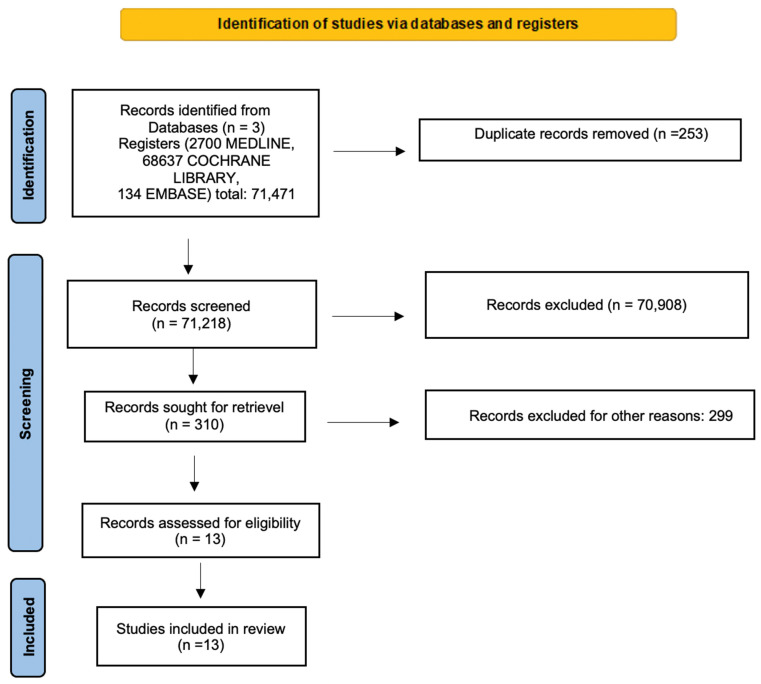
PRISMA (Preferred Reporting Items for Systematic Reviews and Meta-Analyses) flow chart.

The systematic search strategy is provided in Figure 1 (PRISMA). Three different databases were used: PubMed, Embase and Cochrane, with the date of the studies ranging from January 2012 to March 2022.

The database search terms were: (“neoplasms” OR “tumor” AND “radiotherapy” OR “stereotactic radiotherapy” OR “hypofractionation” AND “immune system” OR “lymphocyte” OR “T cells” OR “T lymphocyte” OR “natural killer” OR “NK cells” OR “B lymphocyte”).

### 2.2. Study Selection and Data Extraction

The search was filtered for English language and clinical trial. We identified the titles and the abstracts of 71,471 records on the 11th of March, 2022 with a total of 2700, 68,637, and 134 PubMed, Cochrane and Embase articles, respectively. A manual search, extrapolating articles from the main available meta-analyses, was performed. Furthermore, after removing duplicates and applying “clinical trial” as a filter, each title and abstract was individually screened. Finally, 13 records were selected for full-text reading, which was performed by two independent researchers (S.T., M.B.). Any disagreement was resolved by mutual discussion. We have submitted our review with the PROSPERO ID registration number 356,949.

### 2.3. Inclusion and Exclusion Criteria

Only prospective and retrospective patient cohorts or randomized studies reporting on the effect of stereotactic or hypofractionated treatments on lymphocyte subpopulations in both peripheral blood and tumor microenvironment (TME) were included. Hypofractionated radiotherapy was defined as a dose per fraction ≥2 Gy, while ultra-hypofractionated RT or SBRT was defined as a dose per fraction ≥5 Gy.

Case reports, abstracts, preclinical studies and review papers were excluded. Articles reporting data on lymphocyte subpopulations’ modifications that could not be strictly associated with RT-induced effects, such as RT with other concurrent treatments, were also excluded.

Reference lists of selected publications were further searched for relevant articles. Abstracts were reviewed, and a search for the full publication was performed whenever the topic was relevant, as well as attempts to contact the authors for further details.

## 3. Results

Overall, a total of 13 studies were included in the final analysis: 3 for HFRT [9,10,11] and 10 for SBRT [12,13,14,15,16,17,18,19,20,21]. Only two studies reported on immune cell modification on TME [9,18], analyzing both biopsy tissues and pathological surgical specimens, while the 11 remaining studies analyzed peripheral blood sample [10,11,12,13,14,15,16,17,19,20,21]. Table 1 and Table 2 summarize all relevant details of these studies.

### 3.1. Current Published Evidences of Changes in Lymphocyte Subsets after HFRT

Studies regarding the analysis of immune cells, and in particular lymphocyte subsets modifications, in cancer patients after HFRT are very heterogeneous (Table 1).

In 2018, the LYMPHOREC trial [9] analyzed 237 patients affected by locally advanced colorectal cancer (LARC) undergoing short- or long-course preoperative RT (>2 Gy/fraction and <2 Gy/fraction respectively) and investigated the modulation of immune response before and after RT in tumor tissue. The authors compared tumor-infiltrating-lymphocytes (TILs) on biopsy tissue at baseline and on pathological specimens after RT and surgery, encouraging the routine assessment and quantification of TILs before and after RT to obtain prognostic information as well as deduce potential surrogates of treatment efficacy. Indeed, patients with a significant decrease in the CD8+/Fork-head box P3 (FoxP3+) cells ratio after preoperative RT had a better PFS and OS. Notably, the CD8+/FoxP3+ TILs ratio was significantly lower after short-course than long-course pre-operative RT (*p* = 0.027). The authors highlight that the RT administration schedule affects the CD8+/FoxP3+ TILs ratio and could provide an early indication of treatment effectiveness. 

Yuan et al. [10] compared two fractionation schedules (CFRT: 50 Gy/25 fractions and HFRT: 40.3 Gy/13 fractions) in 60 breast cancer (BC) patients. Lymphocyte count and subpopulations were analyzed on blood samples collected before the radiation treatment, immediately after the last fraction of radiotherapy and 6 months after irradiation therapy ended. They evidenced that HFRT patients had a higher lymphocyte count than patients in the standard fractionation group. The percentage of T CD4+ remained significantly high even after 6 months after treatment ended, while in the conventionally fractionated subgroup, the percentage of T CD4+ cells increased after the last fraction of RT and then dropped to the pre-treatment level after 6 months. There was no significant difference observed in the percentage of T CD8+ cells in the HFRT group, while in the other cohort (25 fractions), this percentage remained unchanged from pre-RT to the last fraction of RT and then dropped significantly 6 months afterwards. Regarding the percentage of B lymphocytes, a decrease after the end of RT was seen in both groups. After 6 months, B cells returned to baseline in the HFRT subgroup, while in the other cohort, they remained lower than the pre-treatment level. Natural killer (NK) cells were not affected by the different fractionation schedule.

In BC, peripheral blood modifications of lymphocyte subpopulations were assessed in a group of low-risk patients undergoing intraoperative RT (IORT) at 48 h, 3 and 10 weeks after RT [11]. Thirteen patients received a 20 Gy-single dose of IORT, and the lymphocyte subpopulations’ phenotyping panel was evaluated. Statistically significant differences in the total number of peripheral blood lymphocytes were not found, but B lymphocytes experienced a decrease after IORT, reaching their lowest value at 10 weeks. The CD4+/CD8+ ratio showed an increase in cytotoxic lymphocytes at 10 weeks as compared to baseline values. The authors also found that the subgroup of NK cells increased significantly 3 weeks after IORT. However, no changes were found in Treg and myeloid-derived suppressor cells (MDSC). Despite the low number of patients evaluated and the short period of immunomonitoring, these preliminary results may support the advantage of this type of HF schedule, which resulted in a minimized risk of immunosuppression after RT.

### 3.2. Current Published Evidence of Changes in Lymphocyte Subsets after SBRT

The studies on the effect of SBRT on the immune system were conducted in patients affected by lung cancer and other type of tumors (Table 2).

Maehata et al. [12] evaluated peripheral blood samples from 62 early stage non-small cell lung cancer (NSCLC) patients before and after SBRT, delivered in 4 to 10 fractions (40–70 Gy). They considered two groups. In the first one, peripheral blood samples were collected before the start of SBRT (pre-treatment) and immediately after SBRT completion (post-treatment). In the second group, blood samples were collected at four different time points: pre-treatment, post-treatment, 1 week and 4 weeks post-treatment. The authors analyzed the change in ALC and lymphocyte subsets (CD3+, CD4+, CD8+, CD19+, CD56+, and NK). The modulation of immune cells was correlated with the clinical follow-up. They observed that all lymphocyte subsets and NK cells at post-treatment and 1 week post-treatment were significantly lower than pre-treatment ones. However, no significant differences in lymphocyte subsets were found among patients with and without relapse.

To unveil this apparent contradiction, the volume of vertebral body (VV) receiving radiation doses of 3, 5, or 10 Gy or more was evaluated. The volume receiving a radiation dose of 3 Gy or more (VV_3_) significantly correlated with all lymphocyte subset changes except for T CD8+ cells. VV_5_ correlated significantly with both ALC and CD19+ changes, while for VV_10_, no correlation was observed for any lymphocyte subsets. However, in contrast with lymphocytes, NK cell variation at 1 week post-treatment did not correlate with any of the dose metrics of the VV, suggesting that NK cells are not a target inside the vertebral body. This study indicates that SBRT, which is considered a low-invasive treatment, is associated with immune system suppression even though it does not correlate with local recurrences, and the decrease in lymphocyte subsets seems to relate to the incidental irradiation of the vertebral bone marrow.

A small group of six patients with stage I NSCLC who were not eligible for surgery and underwent SBRT with a total dose of 48 Gy in 4–8 fractions were analyzed by Zhang et al. [13]. They reported the analysis of peripheral blood immune cell monitoring at three different timepoints: pre-treatment, immediately post-treatment and 3 weeks after the end of RT. The total T cell count increased, particularly CD8+ T cells, while the frequency of CD4+FoxP3+ Treg cells decreased. Other cell subset counts, including CD14+ monocytes and CD3−CD56+ NK cells, did not change significantly after SBRT. The production of some cytokines such as IL-2, TNF-α, and IFN-γ was increased after SBRT, while the suppressive TGF-β in CD4+ T cells was determined to be downregulated. Notably, they observed that peripheral B cell subsets are activated by SBRT. Indeed, the percentages of naïve B cells and double-negative B cells (IgD−CD27−) were decreased after SBRT, while the percentages of marginal zone (MZ)-like B cells, transitional B cells and plasma blast cells increased. These data indicate that SBRT may activate a specific, likely anti-tumor peripheral immune response.

A prospective study of 89 early stage NSCLC patients [14] analyzed peripheral blood T cell counts and the major T cell transcription factors level before and at 2 and 12 weeks after SBRT (54–60 Gy/3–8 fractions). Overall, SBRT was associated with a slight LP that was significantly correlated with the irradiated mean lung dose (MLD), particularly in patients with MLD above 3.7 Gy. However, after SBRT, the proportion of T CD4 and TCD8+ cells, as well as the proportion of T CD4+ cells expressing retinoic acid-related orphan receptor γt (ROR-γt), T-box transcription factor (T-bet) and transacting T–cell-specific transcription factor 3 (GATA-3) increased, while the number of CD4+FoxP3+ Treg cells decreased. The authors suggest that SBRT could enhance the immune system by influencing the systemic immune profile towards an adaptive immune response. On the other hand, SBRT does not induce severe hematologic toxicity, and the risk of LP is correlated with the MLD.

Further evidence of SBRT-mediated activation of the immune system was showed by Navarro et al. [15] in a small cohort of seven lung cancer patients who underwent SBRT (four patients 60 Gy/8 fractions; three patients 50 Gy/4 fractions). The analysis of immune cells from peripheral blood performed before and 72 h after SBRT, and at one, three and six months after SBRT, showed that there was a specific increase in CD56+CD16+NK cells. In parallel, a decrease in the immunosuppressive components of the immune system (CD4+CD25+FoxP3+CDA5RA- Treg cells) and granulocytic- and monocytic-MDSCs was noticed. These changes were still evident six months after treatment. Regarding T lymphocyte subpopulation, cytotoxic T cells (CD3+ and CD8+) showed an increase from baseline to 72 h after SBRT and a decrease at 6 months, while T helper cells (CD3+CD4+) and the CD4+/CD8+ ratio progressively increased from baseline to six months after treatment, with a maximum peak at 3 months.

In an analysis of peripheral blood of a group of 10 patients undergoing liver SBRT (50–60 Gy/3–5 fractions) [16] for both primary and metastatic liver tumors (four patients liver metastases, one patient intrahepatic cholangiocarcinoma, five patients hepatocellular carcinoma), CD3+ T cell counts decreased in all but one patient after SBRT, while CD8+ T cell counts remained unchanged. Contrastingly, the percentage of CD25+CD4+ T cells was reduced, and SBRT did not alter the levels of CD4+CD25+CD127^lo^ Treg cells and appeared to have a differential effect on NK cells. No effect was seen after SBRT for CD56+CD16+ mature cells and cytotoxic NK cells, whereas a decrease was described for CD56brCD16- NK cells, which are less cytotoxic but better able to produce more cytokines. The CD56brCD16- NK cells returned to baseline levels 3 months after treatment. Moreover, SBRT did not affect the surface expression of PD-1 on circulating CD4+ T cells or CD8+ T cells. Interestingly, one patient with liver metastases received pembrolizumab during SBRT, providing the opportunity to evaluate T cell changes when exposed to PD-1 blockade: 71.2% of CD8+ T cells and 55.4% of CD4+ T cells were positive for PD-1 expression in the baseline sample, and as expected, PD-1 levels almost disappeared and remained low in the sample collected. Notably, the authors also examined blood samples from 11 aged-matched healthy volunteers (HV) and found 12 immunophenotypes that differed between patients and HV prior to SBRT treatment, with naïve T cells and circulating dendritic cells (Lineage−HLA-DR+ DCs) being differently represented in the different groups.

In a retrospective study, 78 patients undergoing liver SBRT for hepatocellular carcinoma (HCC) were analyzed (50 Gy/5 fractions for 13 patients, 50–60 Gy/10 fractions for 8 patients, and 48–54 Gy/6 fractions for 57 patients) [17], and ALC and T cell subset changes in peripheral blood before and 10 days after treatment were examined with respect to clinical outcomes such as OS. Frequency of peripheral T cell subpopulations, including CD3+, CD4+, CD8+, CD19+ and CD16+56+ cells, dropped 10 days after SBRT. At univariate analysis baseline and post-treatment, ALC and T cell subset (except for B cells) counts were significantly associated with OS. Indeed, 2 years after treatment, patients with longer survival (>2 years) had a higher level of CD16+CD56+ NK cells compared to those with shorter survival. Significant differences in ALC and CD8+ T cells in patients with long-term and short-term OS at 2 months after SBRT were shown. The authors concluded that peripheral LP after SBRT in HCC patients could be considered as an independent prognostic factor for poorer outcome, and a large PTV was independently associated with an increased risk of LP. Some lymphocyte subsets seemed to be more sensitive to radiation: a marked depletion as well as a slower recovery was shown for B cells (to only 24% of its baseline value) after SBRT than other subpopulations. However, CD3+, CD4+, CD8+ T cells and NK cell counts decreased after SBRT. The increase in CD8+ T and NK cells after treatment were also associated with 2-year OS rates.

In a recent phase I trial, Kane and colleagues [18] investigated pre-treatment biopsies and surgical specimens to determine radiation-induced changes in T cell and macrophage subsets using multiplex immune-fluorescence in six prostate cancer (PCa) patients undergoing neoadjuvant SBRT (24 Gy/3 fractions) 2 weeks before radical prostatectomy. CD8+ T cells were decreased 2 weeks after the end of SBRT, while CD4+T cell and Treg cell (CD4+FoxP3+) densities remained unchanged. The most evident change was the increase in CD11b+ myeloid cells, particularly both the CD68+ and CD163+ macrophage subsets. Moreover, SBRT was associated with a strong alteration of the TME with an integrated, myeloid-centric tissue response that is consistent with RT damage.

McGee et al. [19] analyzed the systemic immune response to SBRT (20–54 Gy/1–5 fractions depending on the irradiated tissue) in peripheral blood of different irradiated organs in 68 patients. Blood samples were obtained prior to RT and 1 or 2 weeks after treatment ended. They observed systemic immune modulation dependent on the irradiated site: no changes after SBRT to non-parenchymal sites (bone, brain); a decrease in total and cytotoxic NK cells; and an increase in T cell immunoglobulin and mucin domain-containing molecule-3-positive (TIM3+) NK cells in parenchymal sites (lung, liver). Notably, total memory CD4+ T cells and activated CD25+CD8+ memory T cells increased after SBRT in parenchymal sites, but not after RT in non-parenchymal sites. 

Crocenzi and colleagues [20] evaluated peripheral blood of 20 pancreatic cancer patients enrolled in two sequential prospective clinical trials of neoadjuvant chemoradiotherapy (CFRT: 50.4 Gy/28 fractions, 10 patients; SBRT: 30 Gy/3 fractions, 10 patients). In this paper, the authors demonstrated that lymphopenia of chemoradiation with CFRT can be bypassed using an hypofractionated regimen and suggested that this is the case of an RT regimen that can be combined with immunotherapies.

Formenti et al. [21] prospectively analyzed tumor tissue and peripheral blood of 39 metastatic NSCLC patients undergoing RT to the metastatic site (30 Gy/5 fx; 28.5 Gy/3 fx) concomitantly with Ipilimumab. The authors found that in peripheral blood, an increase in different immune cells occurred independently of treatment response, with the exception of an increase in PD-1+CD4 T cells occurring in responder patients.

## 4. Discussion

This review provides a unique and detailed overview of the impact of HFRT and SBRT on immune cell modulation in both tumor tissue and peripheral blood samples across different types of tumors.

Hypofractionated RT has gained a crucial role in the treatment of many types of tumors in recent decades, but while its effects in terms of disease control and prolonged survival are well known, its impact on peripheral and tumoral immune cells is not yet clearly defined. Moreover, it is still unclear whether RT-induced immune cell modifications would be correlated with patient recurrence and survival [22,23]. 

Defining the appropriate RT dose able to generate an immune mediated anti-tumor response is of major interest in this new era of radio-immuno combined treatments. The increasing application of SBRT for both localized and disseminated cancers raises interest in the understanding of the immunologic impact of SBRT in this scenario.

Indeed, the combination of local RT, especially high-dose RT, and immune modulation seems to increase local tumor control as well as distant control rates throughout “abscopal” antitumor effects via tumor-antigen release and antigen-presenting cell (APC) cross-presentation, improved dendritic-cell (DC) function and enhanced T cell priming [24]. In many types of tumors treated with HFRT or SBRT, an “abscopal” effect has been reported [25]. The term “abscopal” is used to describe the systemic effect of radiation on “out-of-field” tumor radiation volume [26]. Of note, this effect has been observed when HF or SBRT schedules have been combined with immunotherapy, indicating that this combined modality could be used in daily oncology practice [22,23,24]. However, there is an urgent need to understand which immune cells are modulated and the mechanism underlying the effect of SBRT. In particular, the adequate timing, dose and fractionation necessary to achieve the maximum effect of RT combined with immunotherapy has to be identified. In-depth immunomonitoring considering the immune response modulation at different time points after HF or stereotactic treatment could help to identify the best regimen for this combined strategy.

In this review, we evaluated the role of HFRT and SBRT treatment as potential immune-modulatory strategies as reported in the identified studies, among which only two reported on tumor immune microenvironment modulation [9,18]. In general, HFRT and SBRT are reported to increase the CD8+and CD4+ T cell counts [9,11,12,13,14,19], even in comparison with CFRT [10]. This effect is relevant considering that many studies highlight the importance of CD8+ T cell infiltration and function in complementing the effects of RT [27,28]. Of clinical relevance, the increase in frequency of CD8+ T and NK cells after SBRT has been correlated with OS [17].

While a number of studies found that CFRT generates an increased level of both Treg and MDSC cells, expanding and promoting T cell dysfunction towards immunosuppression, it seems that HF schemes are more advantageous in stimulating the immune system [29]. Considering the effect on TME, in murine models, the comparison of CFRT with HFRT revealed that the latter fractionation schedule may inhibit hypoxia and reduce the recruitment of immunosuppressive cells into primary tumors, generating a microenvironment with lower PD-L1 levels and boosting not only local, but also systemic CD8- mediated anticancer immunity [30]; although, a large number of studies showed that high-dose/fraction irradiation may alter tumor blood vessels, increasing tumor hypoxia with the recruitment of immunosuppressive cells into tumors and a PDL-1 increase [31,32].

Several studies focusing on cancer patients undergoing RT also unveiled the reduction in the absolute number of another subset of lymphocytes, the NK cells [33,34], which represent a critical component in the host surveillance against tumors. The impaired NK cell activity compared to pre-treatment levels suggests that RT directly reduces both NK cell viability and function in a dose-dependent manner. HFRT and SBRT seem to increase an activated NK phenotype, defined by CD56+^high^CD16+, that is related to high cytotoxic activity and low cytokine production [35]. In congruence with their ability to induce tumor regression in allogenic peripheral-blood stem cell transplantation, it has been demonstrated that this T cell subpopulation can induce tumor regression [36]. 

NK-cell-based therapies in pre-clinical and clinical trials are increasingly reported, and treatment strategies including immune-checkpoint blockade using PD-1 or CTLA-4 antibodies have indicated the role of NK cells in the clinical response [37]. It is interesting to note that, in the study of Maehata et al. [12], NK counts at 1 week post-treatment did not correlate with the volume of the vertebral body, demonstrating that, probably in contrast to other analyzed lymphocyte subsets, NK cell count is not influenced by radiation delivered to the vertebral body marrow. 

The function of another group of lymphocytes, B cells, in the tumor microenvironment is controversial. Different B cell subsets play different roles in anti-cancer immunity, and they represent a group of antigen-presenting cells able to activate T cells. Recently, great attention has been paid to the function of B cells, since they are able to generate an integrated cellular and humoral immune response, as demonstrated in renal cancer [38]. However, the dynamics of the changes that occur in B cell composition post-HFRT/SBRT are poorly identified [39].

RT was also shown to generate chemotactic signals that recruit several immune cell types with distinct roles in T cell suppression. Treg cells, expressing FOXP3, are critical to regulate both inflammation and autoimmunity. In the TME, they secrete the cytokines TGFβ and IL-10, which suppress effector T cell activation and stimulate the suppressive functions of MDSCs. Thus, targeting Treg cells and/or their immunosuppressive effector molecules, TGFβ and CTLA-4, could be crucial to reversing immunosuppression [40,41].

After HFRT in LARC [9], the CD8+/FoxP3+ TILs ratio, and in general TILs, were significantly lower compared to CFRT, suggesting that an immunological balance between CD8+ cells’ TILs and FoxP3+ cells’ TILs occur after exposure to HFRT. This decrease correlated significantly with both OS and PFS. Other studies [14,15] in lung cancer showed a decrease of the immunosuppressive component of the immune system after SBRT in peripheral blood. Contrastingly, a prostate cancer trial of neoadjuvant SBRT before radical prostatectomy conducted in only six patients revealed that immune environment was altered 2 weeks after SBRT, with a reduction in CD8+ T cells and an increase in CD68 and CD163 macrophages [18].

Considering the role of immune suppressive or inflammatory cytokines, the inhibitory effect of SBRT on TGF-β in CD4+ T cells was found, while an increase in IL-2, TNF-α and IFN-γ were identified [13].

In the study performed by Gustafson et al. [16], the authors provided evidence, although only in one patient, of changes in T cell subpopulations after treatment with SBRT was combined with PD-1 blockade. Not surprisingly, the PD-1 levels in T cells were strongly downregulated after treatment and maintained this phenotype 3 months after treatment.

In this heterogeneous scenario, the trial by McGee et al. [19] evidenced the relevance of also evaluating whether SBRT were directed to parenchymal or non-parenchymal sites, indicating that the systemic immunological changes are dependent on the irradiated site. No immunological changes occurred when non-parenchymal sites were irradiated. Contrastingly, an increase in TIM3+ NK cells, activated memory CD4+ and CD8+ T cells were found in parenchymal sites, such as lung and liver irradiation sites. The authors suggest that the differences observed may be related to the different dose schemas when these organs are treated. 

There are many limitations in this analysis. Most of the studies were observational and retrospective (only six prospective), and involved small samples of patients of a single institution, with largely different and heterogeneous analyses of immune cell subpopulations among all the studies. Therefore, the overall quality of the evidence gathered is not very high. However, multiple studies testify to the impact of HFRT and SBRT on lymphocyte subpopulations and their role in predicting survival amongst other things in general clinical outcomes.

All the studies identified in this review clearly show that there is a growing interest in both identifying biomarkers in the TME and in the periphery, and employing this knowledge on immunomodulation after SBRT in different tumors and in different sites to clarify how to design future immunotherapy–SBRT clinical trials.

## 5. Conclusions

This review, focused on immunomodulation in the tumor and in the periphery of different hypofractionated RT treatment, suggests the potential immune-stimulatory effect of hypofractionated and stereotactic regimens in different tumors. The preliminary studies highlighted the relevance of in-depth immunomonitoring to better understand SBRT immunomodulation, to define future clinical studies and to better help physicians to develop new combined therapeutic strategies with immunotherapies.

## Figures and Tables

**Table 1:** Hypofractionated radiotherapy studies.

HFRT									
Author	Year	Pathology	n of pts	Study Design	RT	Systemic Therapy	Lymphocyte Parameters	Outcome	Sample
Mirjolet [9]	2018	Rectal Cancer	237	retrospective	NEO-ADJ RT long-course (<2 Gy/fx) vs. short course(>2 Gy/fx)	concomitant CHT + adj	TILs: CD8C and FoxP3 T cells	impact of TILS on PFS and OS: high FoxP3 TIL better PFS; decrease CD8C/FoxP3 TILs ratio better PFS & OS; lower CD8C/FoxP3 ratio with short-course RT	tumor tissue (biopsy and surgical sample)
Yuan [10]	2018	Breast Cancer	60	observational	ADJ RT (50 Gy/25 fx vs. 40.3 Gy/13 fx)	ADJ CHT	TLC, lymph subpopulation (T, B, NK cells)	lymph dropped after RT, recovered at 6 m, higher value in hypo; subpopulations change with different fx schedule	peripheral blood
Linares-Galiana [11]	2021	Breast Cancer	13	observational	IORT (20 Gy/1 fx)	hormone therapy	NK, Treg, MDSC	NK CD56+ high CD16+ increased 3 w after IORT	peripheral blood

Abbreviations: HFRT—hypofractionated radiotherapy; NEO-ADJ—neo-adjuvant; fx—fraction-s; CHT—chemotherapy; TILs—tumor infiltrating lymphocytes; FoxP3—Fork-head box P3; PFS—progression free survival; OS—overall survival; TLC—total lymphocyte counts; lymph—lymphocytes; m—months; fx —fractionation; IORT—intraoperative radiotherapy; NK—natural killer; Treg—T regulatory; MDSC—myeloid-derived suppressor cells.

**Table 2:** Stereotactic body radiation therapy studies.

SBRT									
Author	Year	Pathology	n of pts	Study Design	RT	Systemic Therapy	Lymphocyte Parameters and Cytokines	Outcome	Sample
Maehata [12]	2013	NSCLC	62	retrospective	SBRT (40–70 Gy/4–10 fx)	none	TLC and lymph subsets: CD3+, CD4+, CD8+, CD19+, CD56+, and NKA	Lymph subset, NKA post-RT lower than pre-SBRT	peripheral blood
Zhang [13]	2017	NSCLC	6	observational	SBRT (48 Gy/4–8 fx)	none	T, B cells, cytokines	CD8+ T cells transformed into activated T cells; increase in IL-2, T NF-α, IFN-γ; reduce production of TGF-β in CD4+ T cells; naïve B cells and double-neg B cells lower	peripheral blood
Rutkowski [14]	2017	NSCLC	89	prospective	SBRT (54–60 Gy/3–8 fx)	none	CD4+, CD8+ T cells, T-bet, GATA-3, ROR-γt, FoxP3, CRP, ANC, WBC	CD8+, CD4+, CD4(+) T cells expressing GATA-3+, T-bet+, ROR-γt+ increased; CD4+FoxP3+ cells decreased	peripheral blood
Navarro [15]	2018	NSCLC, LUNG M+	7	prospective	SBRT (50–60 Gy/4–8 fx))	none	total lymph, CD56+high CD16+NK, CD4+CD25+ Foxp3+ CDA5RA Treg, G-MDSCs, Mo-MDSCs	Increase CD56+highCD16+NK 6 m; decrease CD4+CD25+ Foxp3+CDA5RA Treg, G-MDSCs and Mo-MDSCs at 6 m; T CD3+CD8+, T CD3+CD4+ and TCD4/CD8 ratio increase	peripheral blood
Gustafson [16]	2017	Liver cancer (CCA, HCC, liver M+)	10	observational	SBRT (50–60 Gy/3–5 fx)	none	CD8+ T cells; CD4+CD25+ CD127lo Treg cells; CD56+CD16+ NK cells; PD-1	no difference in CD8+ T cells, CD4+CD25+ CD127lo Treg cells, NK cells and PD-1 expression; decrease in CD56brCD16- NK	peripheral blood
Zhuang [17]	2019	HCC	78	Retrospective	SBRT (48–60 Gy/5–8 fx)	none	TPLCs, CLPs	TPLC, B cells, NK, T cells subpop reduced 10 days after SBRT (B cells lower value)	peripheral blood
Kane [18]	2022	PCa	6	prospective	neoadj SBRT (24 Gy/3fx) (prior to RP)	none	T CD3+CD8+, T CD3+CD4+, Treg cell (CD4 + FOXp3), CD68 + and CD163+ macrophage	T CD3+CD8+ decreased; T CD3+CD4+ and Treg cell (CD4 + FOXp3) stable; CD68 and CD163+ macrophage increased	tumor tissue (biopsy and surgical sample)
McGee [19]	2019	various: lung, liver, adrenal, brain, bone, and others	68	prospective	SBRT (20–54 Gy/1–5 fx)	none	CD8 T cells, CD4 T cells, NK cells, TIM3+ expression	Total and cytotoxic NK decreased, TIM3+ increased, CD4+ T cells, activated and CD25+ CD4+ memory T cells, and activated CD25+ CD8+ memory T cells increased, TNF-a, RANTES decreased after RT in parenchymal sites, no brain	peripheral blood
Crocenzi [20]	2016	Pancreatic Cancer	20	prospective	SBRT (30 Gy/3 fx) vs. CFRT (50.4 Gy/28 fx)	NEO-ADJ CHT	T cells subsets: CD3+, CD4+, CD8+, Treg; cytokines;	CD4+, CD8+, Treg reduced in CFRT. IL-15 reduced in CFRT and SBRT	peripheral blood
Formenti [21]	2019	NSCLC M+	39	prospective	SBRT (30 Gy/5 fx; 28.5 Gy/3 fx)	IT	T cells subsets (peripheral bl): CD3+, CD4+, CD8+, Treg;	increase in CD8+, CD4+ T cells	peripheral blood

Abbreviations: SBRT—stereotactic body radiotherapy; NSCLC—non-small cell lung cancer; FoxP3+—Fork-head box P3; TLC—total lymphocyte counts; lymph—lymphocytes; NKA—natural killer cell activity; fx—fraction-s; CRP—Serum C-reactive protein; ANC—absolute neutrophil count; ALC—absolute lymphocyte count; WBC—white blood cell; CCA—cholangiocarcinoma; HCC—hepatocellular carcinoma; fx—fractionation; G-MDSCs—granulocytic myeloid-derived suppressor cells; Mo-MDSCs—monocytic myeloid-derived suppressor cells; m—months; TPLCs—total peripheral lymphocyte counts; CLPs—circulating lymphocyte population; CFRT—conventional fractionated radiotherapy; NEO-ADJ CHT—neo-adjuvant chemotherapy; IT—immunotherapy; PDL-1—programmed death ligand-1.
